# Mineral and Bone Disease in Hemodialysis Patients

**DOI:** 10.5812/numonthly.9510

**Published:** 2013-09-15

**Authors:** Paraskevi Theofilou

**Affiliations:** 1 Centre for Research and Technology, Department of Kinesiology, Health and Quality of Life Research Group, Thessaly, Greece

**Keywords:** Renal Insufficiency, Chronic, Renal Dialysis, Bone Diseases

Dear Editor,

I have read with interest the article by Sidy Mohamed Seck et al. titled “Mineral and Bone Disease in Black African Hemodialysis Patients: A Report from Senegal” published in Nephro-Urology Monthly (2012; 4(4): 613-616) (1). As pointed out in the article, chronic kidney disease related mineral and bone disease (CKD-MBD) is frequent in Senegalese hemodialysis patients and is dominated by high turn - over disease. It is essential to refer that this study is a great contribution not only to the quality of the journal but also to the scientific community. Although a considerable number of articles on CKD have been published, there are a limited number of studies with regards to CKD-MBD, especially in Africa.

The writing is concise; the aim of the study is very clear as well as the study is very - well designed. The authors have used relative and up - to - date bibliography comparing in a comprehensible way their results to other studies’ findings. Further, the present study is a first step in identifying the need of the implementation of an intensive educational program, with a multi - faceted approach, focused on changing health professional behavior in managing patients, with small but clinically relevant improvements (2). Educational interventions, comprising different types of activities intended to increase the knowledge and skills of healthcare professionals and patients, have traditionally been the predominant approach to stimulate change and improvement in healthcare (3). I would like to congratulate the authors of this paper and wish them every success in their ongoing research.
